# Evaluation of engineered AAV capsids for hepatic factor IX gene transfer in murine and canine models

**DOI:** 10.1186/s12967-017-1200-1

**Published:** 2017-05-01

**Authors:** David M. Markusic, Timothy C. Nichols, Elizabeth P. Merricks, Brett Palaschak, Irene Zolotukhin, Damien Marsic, Sergei Zolotukhin, Arun Srivastava, Roland W. Herzog

**Affiliations:** 1 Department of Pediatrics, University of Florida, Gainesville, FL 32610 USA; 2 Department of Pathology and Laboratory Medicine, University of North Carolina-Chapel Hill, Chapel Hill, NC 27599 USA

**Keywords:** AAV, Gene therapy, Hemophilia B, Factor IX, Capsid

## Abstract

**Background:**

Adeno-associated virus (AAV) gene therapy vectors have shown the best outcomes in human clinical studies for the treatment of genetic diseases such as hemophilia. However, these pivotal investigations have also identified several challenges. For example, high vector doses are often used for hepatic gene transfer, and cytotoxic T lymphocyte responses against viral capsid may occur. Therefore, achieving therapy at reduced vector doses and other strategies to reduce capsid antigen presentation are desirable.

**Methods:**

We tested several engineered AAV capsids for factor IX (FIX) expression for the treatment of hemophilia B by hepatic gene transfer. These capsids lack potential phosphorylation or ubiquitination sites, or had been generated through molecular evolution.

**Results:**

AAV2 capsids lacking either a single lysine residue or 3 tyrosine residues directed substantially higher coagulation FIX expression in mice compared to wild-type sequence or other mutations. In hemophilia B dogs, however, expression from the tyrosine-mutant vector was merely comparable to historical data on AAV2. Evolved AAV2-LiC capsid was highly efficient in hemophilia B mice but lacked efficacy in a hemophilia B dog.

**Conclusions:**

Several alternative strategies for capsid modification improve the in vivo performance of AAV vectors in hepatic gene transfer for correction of hemophilia. However, capsid optimization solely in mouse liver may not predict efficacy in other species and thus is of limited translational utility.

**Electronic supplementary material:**

The online version of this article (doi:10.1186/s12967-017-1200-1) contains supplementary material, which is available to authorized users.

## Background

Adeno-associated viral (AAV) vectors represent some of the most powerful tools for in vivo gene transfer to a variety of organs. For example, impressive successes with ocular gene transfer have been reported, resulting in reversal of inherited forms of blindness [1]. AAV vectors have also been in clinical development for hepatic gene transfer over the past decade, primarily to express coagulation factors in hepatocytes for the treatment of the X-linked bleeding disorder hemophilia. Most recently, curative levels of factor VIII (FVIII) and factor IX (FIX) were achieved in patients with hemophilia A or hemophilia B, respectively [2, 3]. This was achieved using viral capsids derived from several different serotypes. However, in some cases very large vector doses were required. The viral capsid is a major determinant of AAV tropism, and some capsids have not performed as efficiently in humans as they did in preclinical studies. In particular, transduction of mouse liver has been more effective than human liver. Thus, the search for optimal serotypes/capsids for transduction of human hepatocytes continues, for instance through studies in non-human primates or in “humanized” mice with chimeric mouse–human livers [4, 5]. Another obstacle that continues to negatively affect gene therapy for hemophilia is the activation of capsid-specific CD8^+^ T cells [6]. Hepatocytes cross-present the viral input capsid antigen via MHC I, following endosomal escape of the infecting viral particles, ubiquitination, and proteasomal degradation [7–9]. Capsid-specific cytotoxic T lymphocytes can target AAV transduced hepatocytes, resulting in loss of expression and toxicity [10, 11]. Immune suppression regimens have been developed to counter these T cell responses [12, 13].

Enhancing the performance of AAV gene transfer could potentially result in therapy at lower vector doses, which should also reduce capsid antigen presentation. It is hoped that reducing capsid antigen below a threshold level will resolve this issue. We sought to develop vectors that are effective at lower vector doses and postulated that AAV capsids could be engineered to alter intracellular trafficking post-entry of the vector into target cells, thereby enhancing delivery to the nucleus. Our studies of the life cycle of AAV2 demonstrated that shunting of viral particles to the proteasome and subsequent degradation is promoted by phosphorylation of capsid tyrosine (Y) residues, which provide a signal for capsid polyubiquitination [14]. Therefore, we hypothesized that substitution of surfaced exposed tyrosine residues in the AAV2 capsid would significantly reduce proteasomal processing of capsid. Thus, we individually mutated seven surface exposed tyrosine residues at positions (252, 272, 444, 500, 700, 704, and 730) to phenylalanine and observed enhanced in vitro transduction at residues 444, 500, and 730, that was not augmented by either tyrosine kinase or proteasome inhibition. These mutant capsids showed a reduction in polyubiquitination and directed increased trafficking to the nucleus [15]. The combination of Y444F, Y500F, and Y730F mutations (AAV2-(Y-F)-M3) resulted in the best improvement in vitro, and in vivo for murine hepatocyte transduction [16]. As immunological presentation of capsid antigen is dependent on proteasome degradation, the M3 mutation protected AAV transduced human hepatocytes in vitro and murine hepatocytes in vivo from CD8^+^ T cell targeting and killing, thus validating our hypothesis [9].

Besides surface-exposed tyrosine residues, serine and threonine residues represent potential phosphorylation sites on the AAV capsid. Elimination of lysine residues may directly prevent ubiquitination, as opposed to the indirect method of preventing capsid phosphorylation, which serves as a signal for ubiquitination [17]. After identifying an optimal combination of Y-F variants for hepatocyte gene transfer, we therefore sought to determine if substitution of alternative kinase targets serine (S) and threonine (T) and ubiquitin target lysine (K) would further improve transduction [18, 19]. In an alternative strategy, we developed a combinatorial library, initially based on the AAV2 *cap* sequence, with an unprecedented complexity to select for capsid variants that optimally infect hepatocytes [20, 21]. As proof-of-principle, we selected for two variants, termed AAV2-LiA and AAV2-LiC, with high tropism for murine liver [20]. Here, we evaluated AAV2-LiA and AAV2-LiC variants for correction of murine hemophilia B by hepatic gene transfer. Further we compared several engineered AAV2 and AAV8 capsid variants with Y, S, T, and L substitutions for in vivo *F9* gene transfer to mouse liver. Finally, AAV2-(Y-F)-M3 and AAV2-LiC were tested in a large animal model, hemophilia B dogs, for FIX expression following liver-directed gene transfer.

## Methods

### AAV vector construction and production

Site-directed mutagenesis and plasmids pACGr2c2 and pACGr2c8 was performed as previously described using a two-stage PCR reaction [15, 18, 19]. AAV2-LiA and AAV2-LiC were identified by sequencing DNA isolated from liver tissue following three successive rounds of in vivo capsid library infection as previously described by Marsic et al. [20]. AAV vectors expressing human *F9* from the liver-specific ApoE enhancer and α_1_-antitrypsin promoter were packaged into AAV2, AAV2-LiA, AAV2-LiC, AAV2 variants (Table 1), AAV8, and AAV8 variants (Table 1) capsids as previously published [16].

**Table 1:** Summary of AAV variants and mouse strains tested

Serotype	(Y-F)	(T-V)	(K-E)	Mouse
AAV2-(Y-F)-M3	444, 500, 730			C57BL/6
AAV2-(Y-F)-M3-T491V	444, 500, 730	491		C57BL/6
AAV2-(Y-F)-M3-(T491+550V)	444, 500, 730	491 and 550		C57BL/6
AAV2-K544E			544	C57BL/6
AAV2-K556E			556	C57BL/6
AAV2-LiA	444 and 500			BALB/c-HB
AAV2-LiC	500			BALB/c-HB
AAV8				C57BL/6
AAV8-T494V		494		C57BL/6
AAV8-(Y275+447+733F)	275, 447, and 733			C57BL/6

### Experimental animals

Murine studies were approved under University of Florida Institutional Animal Care and Use Committee protocol #201503182. Male hemophilia B mice on a BALB/c background (BALB/c-F9^−/Y^) are bred onsite and have a targeted deletion of the murine *F9* gene [22, 23]. Male C57BL/6 mice were purchased from The Jackson Laboratory (Bel Harbor, ME). All studies were conducted in mice 6–8 weeks old. Vector was infused by tail injection at either 1 × 10^10^ or 1 × 10^11^ vg as indicated. When indicated, BALB/c-F9^−/Y^ mice received a subcutaneous immunization of 1 IU recombinant FIX protein in adjuvant (Sigma Adjuvant System S6322). Blood samples for BALB/c-F9^−/Y^ mice were collected by tail transection into 3.8% citrate buffer (0.38% final). For all C57BL/6 studies mice were bled from the retro-orbital plexus into heparinized micro-capillary tubes.

A canine hemophilia B dog colony is maintained at the Francis Owen Blood Research Laboratory (University of North Carolina at Chapel Hill). These animals have a missense mutation (E379G) in canine factor IX that results in misfolded cFIX protein and complete absence of cFIX protein in circulation. All experiments were conducted in male hemophilia B dogs that were pre-screened for anti-AAV neutralizing antibodies prior to enrollment. All animals were housed in US Department of Agriculture approved facilities and the experimental protocol was approved by the Institutional Animal Care and Use Committee at UNC-Chapel Hill. The weights and ages of animals at the time of vector infusion are listed in Table 2. Plasma was collected per study protocol and shipped to UF. CBC, WBCT, and other routine blood tests were performed onsite at UNC-Chapel Hill on a scil Vet abc cell counter calibrated for canine cells. Vector was infused by portal vein (AAV2-(Y-F)-M3) and saphenous vein (AAV2-LiC) at the indicated vector doses as published [24].

**Table 2:** Overview of hemophilia B canine studies

Dog	Serotype	Route	Dose (vg/kg)	Weight (kg)	Age (year month)	Gender
O19	AAV2(Y-F)-M3	PV	2 × 10^11^	27.8	2 year 1 month	Male
O58	AAV2(Y-F)-M3	PV	6 × 10^11^	23.2	2 year 6 month	Male
Bruce	AAV2-LiC	IV	6 × 10^11^	21.4	4 year 1 month	Male

### Blood/plasma analysis or FIX, coagulation, and antibody assays

Plasma samples from hemophilia B mice and dogs were analyzed using a modified activated partial thromboplastin time assay (aPTT). The %hFIX activity in mice or %cFIX activity in dogs was determined from a log–log standard curve generated from dilutions of normal human and dog plasma. Inhibitory antibodies to hFIX or cFIX were measured by Bethesda assay as previously described [25, 26]. One Bethesda unit is defined as the reciprocal of the dilution of test plasma at which 50% of cFIX or hFIX activity is inhibited. Measurements were carried out on a Diagnostica Stago STart Hemostasis Analyzer (Parsippany, NJ). Levels of hFIX antigen in mice and IgG1 antibodies to FIX were measured by enzyme-linked immunosorbent assay (ELISA) as previously described [25]. Levels of cFIX antigen in mouse plasma were measured by ELISA (Affinity Biologicals CFIX-EIA) using a standard derived from normal pooled canine plasma following the manufacturer’s protocol.

### Detection of AAV NAB

Neutralizing antibodies (NABs) against AAV2(Y-F)-M3 or AAV2-LiC vector particles were measured by inhibition of in vitro GFP transduction of HeLa cells and reported as the reciprocal dilution of plasma that resulted in 50% inhibition of transduction relative to control cells transduced with virus alone. Cells were co-infected with adenovirus to enhance AAV mediated GFP expression.

### Statistical analysis

Results are presented as mean ± SD. Differences between experimental groups were evaluated using one-way ANOVA with Turkey multiple comparisons test or unpaired T tests when only two groups were compared.

## Results

### Lysine mutant capsid as potential alternative to tyrosine mutations for improved factor IX expression

Previous reports have shown that elimination of surface exposed lysine residues, a putative site for polyubiquitination, on AAV2 leads to an increase in murine hepatocyte transduction efficiency over wild-type capsid [19, 27]. Yet a limitation of these studies was the use of a non-secreted transgene product, GFP or luciferase, that hindered quantitative comparison of transgene expression and did not account for systemic delivery as needed in the treatment of hemophilia. Secondly, some of these studies were conducted in reference to wild-type AAV2 and lack direct comparison to the optimized AAV2(Y-F)-M3 capsid [16]. In the present study, we evaluate the efficacy of two single (K-E) substitutions at positions 544 or 556 compared against both AAV2 and AAV2(Y-F)-M3 vectors in C57BL/6 mice using a ssAAV-ApoE-hAAT-*hF9* vector (expression from the ApoE hepatocyte control region and enhancer, combined with human α_1_-antitrypsin promoter) that expresses human factor IX protein (hFIX) from a liver specific promoter summarized in Table 1. Although a K to R substitution conserves the amino acid charge, in our hands we were unable to generate suitably infectious viral particles [19, 27].

**Fig. 1:**
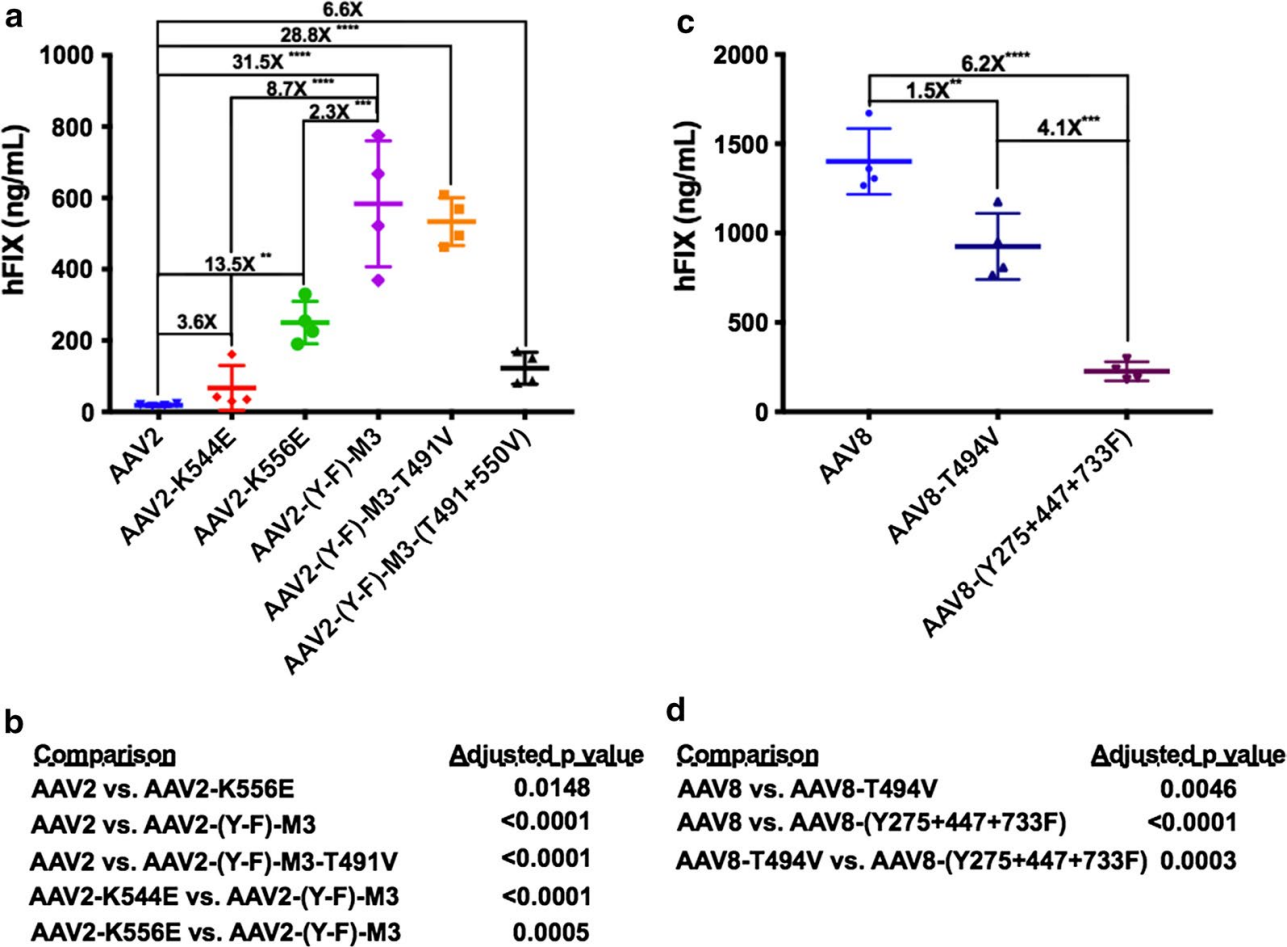
In vivo evaluation of murine liver gene transfer in wild-type AAV2 and AAV8 vectors and respective variants. Male C57BL/6 mice (n = 4 per group) were injected by tail vein with 1 × 10^10^ vg of an AAV vector (ApoE-hAAT-*hF9*) expressing human FIX protein from a liver specific promoter. hFIX levels in plasma was determined by ELISA at 4 weeks following vector injection for **a** AAV2 and variants with **b** statistical summary and **c** AAV8 and variants with **d** statistical summary. The specific Y, T, and K mutations of each capsid are summarized in Table 1. Relative fold difference between groups is listed above selected groups and the amount of “*” included is as reported by one-way ANOVA Tukey multiple comparisons test

Mice were injected in the tail vein with 1 × 10^10^ vg of each vector, and hFIX levels were determined in plasma by ELISA at 1 month after gene transfer. On average, AAV2-K544E yielded a 3.6-fold increase in systemic hFIX expression compared to AAV2, although not reaching a statistically significant difference. In contrast, AAV2-K556E treated mice had a significant and more robust increase in levels of circulating hFIX protein compared to AAV2 (13.5-fold, Fig. 1a). These did not quite reach the levels of AAV2(Y-F)-M3 treated mice, which expressed 2.3-fold higher hFIX as compared to AAV2-K556E transduced mice. Hence, K556 may be a critical site for AAV2 capsid ubiquitination, and elimination of this single lysine residue increases transgene expression to near the levels achieved with optimal multiple tyrosine mutant capsid.

Next, we asked if a single (T491V) or double (T491V and T550V) threonine mutants combined with AAV2-(Y-F)-M3 would further enhance murine hepatocyte gene transfer, as has been suggested in prior work [18]. In contrast to those earlier data obtained with non-secreted reporter genes, we did not observe an improvement in hFIX levels with the addition of either T mutation (Fig. 1a). AAV2-(Y-F)-M3 injected mice expressed 31.5-fold more hFIX compared to wild-type AAV2, consistent with our previously published findings (Fig. 1a) [16].

### Inability to further improve AAV8 performance in murine liver

Among naturally occurring serotypes, AAV8 has the highest transduction efficiency of murine liver. We have recently shown that substitution of surface exposed lysine (K) residues in AAV8 did not lead to a substantial improvement in murine liver transduction and in some instances, was detrimental [19]. Therefore, we tested if instead, substitution of surfaced exposed tyrosine (Y) or threonine (T) residues could further improve hepatic AAV8 gene transfer in mice. AAV2 and AAV8 amino acid alignment and surface residue prediction resulted in the identification of AAV8 Y residues 275, 447, and 733 and a T residue at position 494, which maps closely to those identified in AAV2, excluding Y-275. However, neither AAV8(Y-F)-M3 nor AAV8-T494V vectors improved hFIX expression levels compared to wild-type AAV8 (Fig. 1b), similar to our previous findings with lysine substitutions.

### Partial correction of canine hemophilia B with AAV2-(Y-F)-M3 vector

An advantage in development of novel therapies for hemophilia is the existence of a well characterized large animal model, in form for hemophilic dogs [28, 29]. Of the various Y, K, S, and T mutation capsids, AAV2-(Y-F)-M3 showed the strongest improvement in hFIX expression in mouse liver compared to the parent capsid, in this case AAV2. Previously, several hemophilia B dogs injected in the mesenteric or portal vein with an AAV2-(ApoE)_4_-hAAT-*cF9* vector, expressing canine FIX (cFIX), at doses of approximately 1 × 10^12^ vg/kg achieved sustained therapeutic levels of ~5% of normal for multiple years [30, 31]. Based on these studies, we elected to test AAV2-(Y-F)-M3 in the canine model. We constructed a vector that expresses cFIX from the ApoE/hAAT combination used in the mouse experiments described above (thus similar to the hAAT promoter combined with 4 copies of the ApoE enhancer in the previously published studies). The vector was to be administered to male hemophilia B dogs from the UNC-Chapel Hill colony. These dogs have a missense mutation, leading to expression of cFIX in hepatocytes but lacking circulating cFIX antigen and activity [32]. All prospective subjects were screened for lack of AAV capsid neutralizing antibodies using an in vitro neutralizing antibody (NAB) assay. Since in mice we observed a significant enhancement in hFIX expression following portal vein delivery of AAV(Y-F) mutant vector compared to peripheral vein injection [15], we chose to administer the AAV2-(Y-F)-M3 vector via the portal vein.

**Fig. 2:**
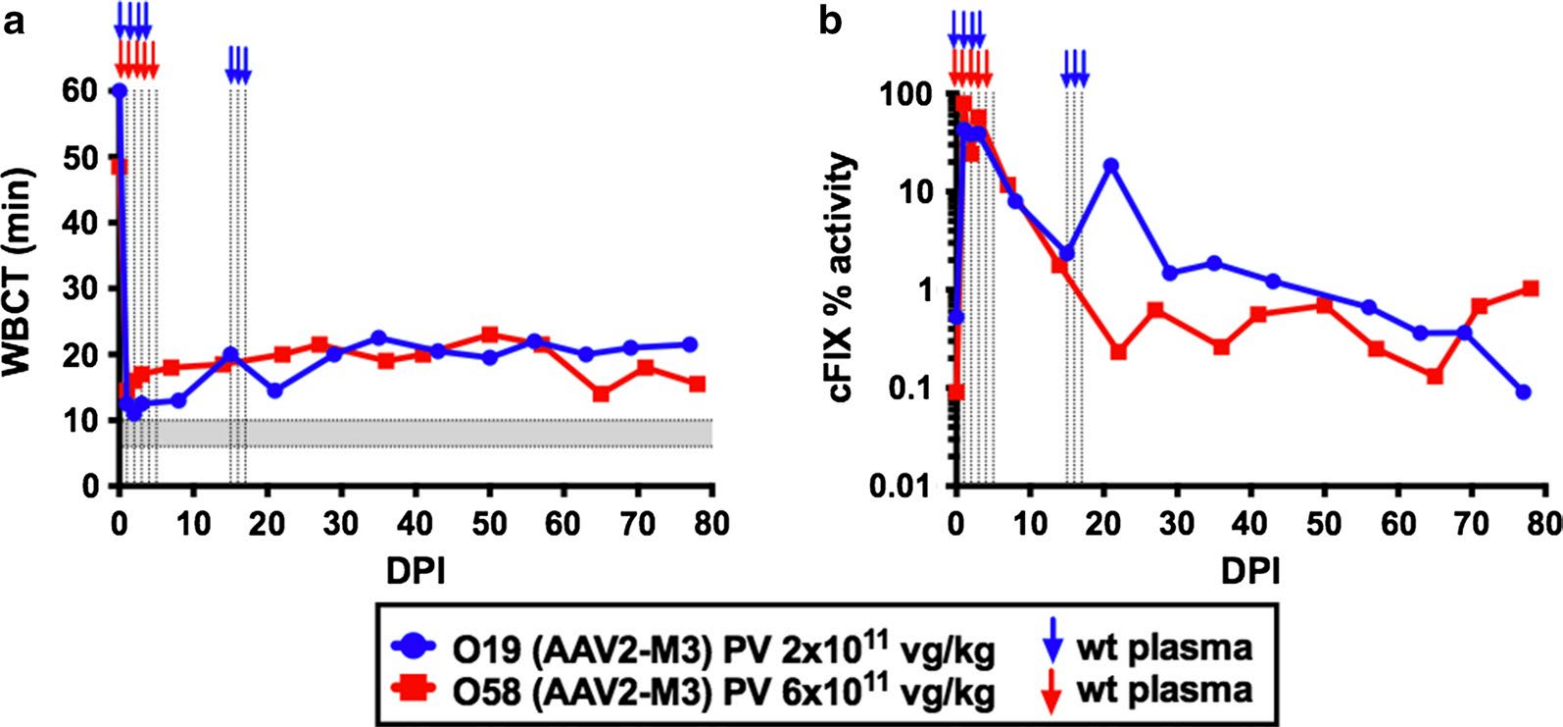
Hemophilia B dogs injected with two different doses of AAV2-(Y-F)-M3-ApoE-hAAT-*cF9* vector do not express therapeutic levels of cFIX protein. **a** Whole blood clotting times (WBTC) and **b** cFIX activity of dogs O19 (*blue* 2 × 10^11^ vg/kg) and O58 (*red* 6 × 10^11^ vg/kg) over time. *Colored arrows* indicate infusion of wild-type dog plasma. Normal WBCT is indicated by *shaded gray* area on graph

Two male dogs O19 2.1 years and O58 2.5 years were chosen, see Table 2 for details. The first animal, O19, was injected at a vector dose of 2 × 10^11^ vg/kg, approximately fivefold lower than historically treated AAV2 dogs, into the portal vein [24]. To cover the animal during the surgical intervention, O19 received coverage with normal dog plasma with a total of 450 mL on the day of injection followed by 300 mL on days 1, 2, and 3 post-injection. A hematoma was noted at the injection site on day 15 post-injection, and 150 mL plasma was administered on days 15, 16, 17, and 18. As a result of gene transfer, WBCT fell from pre-treatment of greater than 60 min to a mean of 21.8 ± 2.2 min (calculated from day 29 onward) post gene therapy (Fig. 2a, blue tracing). Canine FIX activity, measured over a period of 97 days post treatment, ranged from 0.3 to 1.5% of normal. At study endpoint, cFIX activity was approximately 0.5% of normal (Fig. 2b, blue tracing). Clinical chemistry and CBC revealed a moderate reduction in platelet counts over the course of the study and a mild to moderate elevation in ALT prior to treatment and towards the end of the study on days 97, 140, and 175 (Additional file 1: Table S1).

A second animal, O58, was injected with a threefold higher vector dose of 6 × 10^11^ vg/kg and was followed over time. Dog O58 received 800 mL normal plasma over the course of the day of injection, followed by 350 mL on day 1, 400 mL day 2, and 200 mL on days 3, 4, and 5. There were no major complications or bleeds following vector injection. Clinical chemistry and CBC counts revealed a mild reduction in platelets and a slight elevation in hematocrit and hemoglobin (Additional file 2: Table S2). At this higher vector dose, WBCTs decreased from a pre-treatment value of 48 min to an average of 19.1 ± 2.6 min (Fig. 2a, red tracing). A cFIX activity of around 1% of normal was achieved (Fig. 2b, red tracing), when the WBCT was 14-18 min. As expected for hepatic gene transfer to these animals with a *F9* missense mutation, gene transfer did not induce an inhibitor against cFIX in either dog (Additional file 3: Figure S1).

### Treatment of murine and canine hemophilia B using capsids evolved to optimally transduce murine liver

**Fig. 3:**
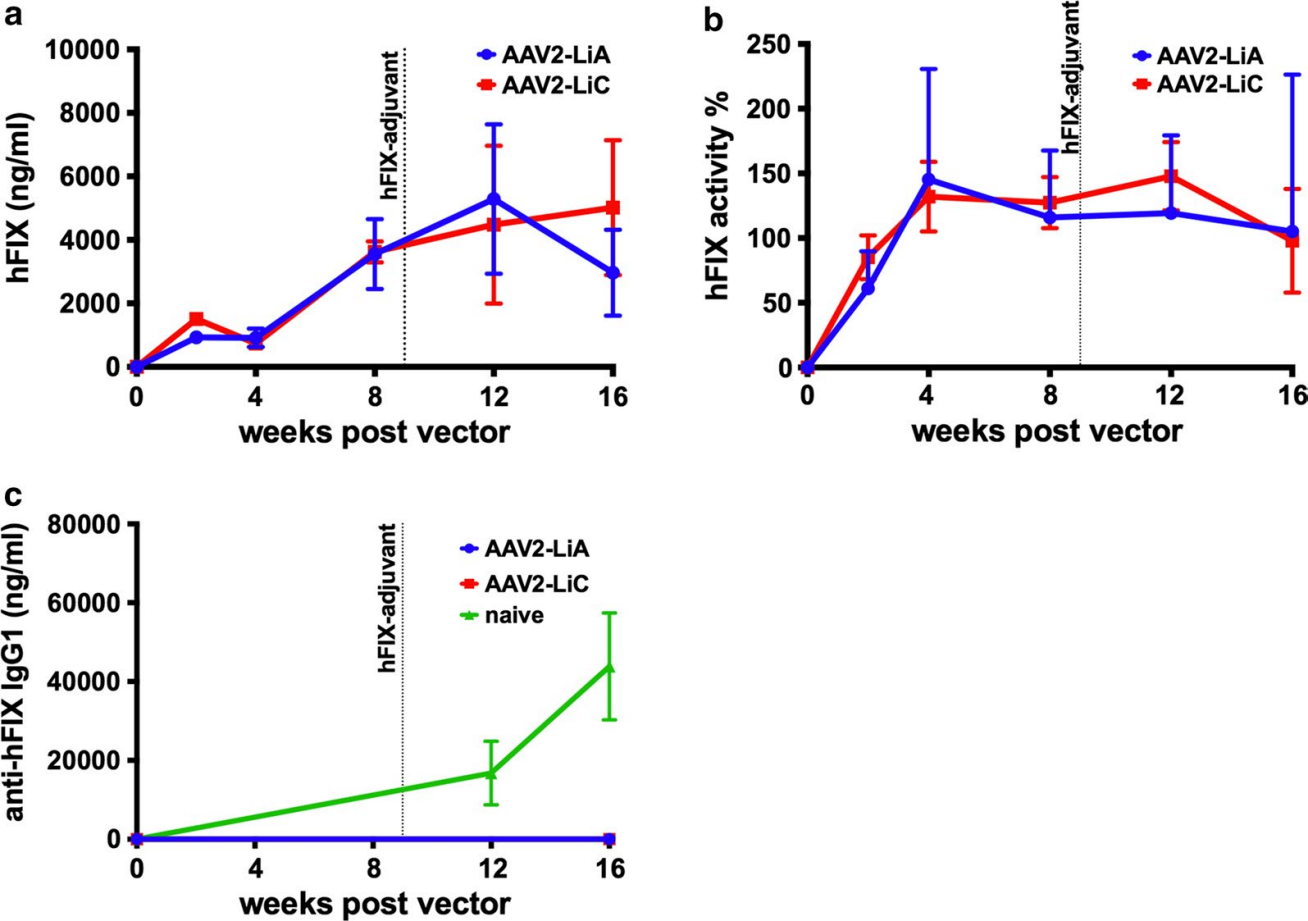
AAV2-LiC capsid selected for murine hepatocyte tropism normalizes coagulation and induces tolerance in hemophilia B mice. Hemophilia B mice (BALB/c-F9^−/Y^) were intravenously injected with 1 × 10^11^ vg of either AAV2-LiA or AAV2-LiC ApoE-hAAT-*hF9* vector and followed over time for **a** hFIX protein levels **b** % FIX coagulation activity, and **c** anti-hFIX IgG1 antibody titers. *Horizontal dotted line* in **a**–**c** denotes time of adjuvant-hFIX challenge in mice

Next, we investigated the efficacy of two AAV2 variants that were selected for murine hepatocyte tropism through repetitive rounds of screening a rationally designed capsid library [20]. AAV2-LiA contains two Y-F substitutions at 444 and 500 in addition to 12 additional mutations, while AAV2-LiC has only a single Y-F substitution at position 500 and 3 additional mutations (Table 1) [20]. Unlike AAV2-(Y-F)-M3, both AAV2-LiA and AAV2-LiC serotyped vectors are equivalent to AAV8 for hFIX expression in mouse liver, providing comparable levels following intravenous delivery. Therefore, we tested the efficacy of both AAV2-LiA and AAV2-LiC in hemophilia B mice (BALB/c-*F9* ^−/Y^) injected at a dose of 1 × 10^11^ vg with ssAAV-ApoE-hAAT-*hF9* vector. Mice were followed over time for hFIX protein levels by ELISA, and activity levels were derived from a standard curve in an aPTT assay. Mice treated with either vector expressed comparable levels of hFIX protein and had similar high activity, which reached normal to super physiological levels of up to ~150% (Fig. 3a, b). Nine weeks following vector injection, vector treated and naïve mice were immunologically challenged with 1 IU of recombinant hFIX protein in adjuvant. None of the animals treated with gene therapy developed antibodies against hFIX or had a reduction in circulating hFIX protein or coagulation activity (Fig. 3a–c), while naïve challenged BALB/c-*F9* ^−/Y^ mice developed robust anti-FIX titers. Thus, hepatic gene transfer had induced humoral tolerance to hFIX.

**Fig. 4:**
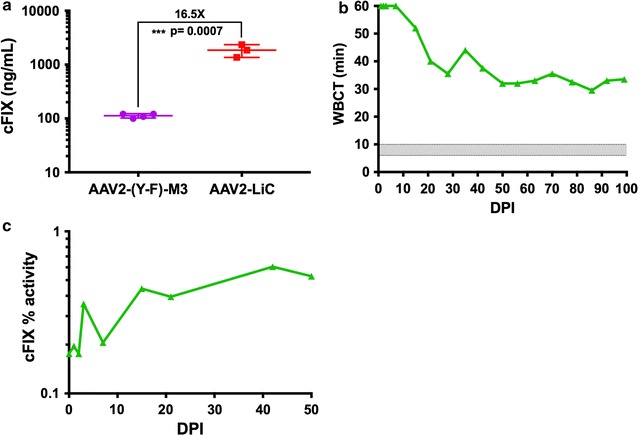
Administration of AAV2-LiC-ApoE-haat-*cF9* to a hemophilia B dog leads to only minimal correction of coagulation. **a** C57BL/6 mice were intravenously injected with 2 × 10^10^ vg of the AAV2-(Y-F)-M3-ApoE-hAAT-*cF9* vector or 1 × 10^10^ vg of the AAV2-LiC-ApoE-hAAT-*cF9* and levels of circulating cFIX protein were measured by ELISA at 4 weeks post gene delivery. **b**, **c** A hemophilia B dog, Bruce, was peripherally injected with 6 × 10^11^ vg/kg of an AAV2-LiC-ApoE-hAAT-*cF9* vector and followed over time for changes in **b** WBCT and **c** % cFIX activity. Fold difference between groups in **a** are listed above and the amount of “*” and p value is included as reported by an unpaired T test

To test one of these capsids, which were highly efficient in hemophilic mice, in the hemophilia B dog model, we produced AAV2-LiC-ApoE-hAAT-*cF9* vector. We confirmed in mice that the vector directed high levels of cFIX expression (~30-fold higher compared to the AAV2-(Y-F)-M3 vector when normalized to vector dose, Fig. 4a). Three prospective animals were screened for the presence of pre-existing NAB against AAV2-LiC, and all three were negative. Based on previous outcomes with AAV8 vectors in hemophilia B dogs [33], and comparable gene transfer efficiency of AAV2-LiC with AAV8 in mice [20], we selected a vector dose of 6 × 10^11^ vg/kg with intravenous delivery into our subject (hemophilia B dog Bruce, 4.1 years, Table 2). Clinical chemistry and CBCs did not reveal any abnormalities over the course of the study, results are presented in Additional file 4: Table S3. However, Bruce achieved only a modest decrease in WBCT from >60 min to an average of 34.4 ± 3.8 min (Fig. 4b), without evidence for inhibitor formation (Additional file 3: Figure S1). As expected from the WBCTs, cFIX activity was consistently <0.5% of normal (Fig. 4c). Thus, the high efficiency of FIX expression in hepatic gene transfer from peripheral vein in mice did not translate into efficacy in hemophilic dogs.

## Discussion

Current gene therapy trials for the treatment of hemophilia A and B are all based on liver-directed gene transfer with AAV vectors injected into a peripheral vein [2, 3]. Different serotypes with liver tropism are used to transduce hepatocytes. Major advantages of liver gene transfer include the capacity of hepatocytes to efficiently secrete proteins into the circulation, their ability to perform the posttranslational modifications that are needed to produce biologically active FVIII and FIX, and induction of immune tolerance to the hepatocyte-expressed transgene product [34]. Current obstacles are the high vector doses that are typically required and activation of capsid-specific CD8^+^ T cells. Both problems can potentially be addressed by engineering the capsid. Informed by an improved understanding of the biology of the viral interactions with target cells, specific mutations can be designed to eliminate amino acids that promote proteasomal degradation of capsid, thereby limiting efficacy and increasing MHC I antigen presentation [17].

### Empirical evaluation of candidate mutations reveals alternative classes of amino acid changes that improve performance of different serotype capsids

With the goal of eliminating either capsid phosphorylation or ubiquitination, we tested a variety of amino acid changes. Combinations of these may have synergistic effects. As of now, there is no method to predict their effects. Thus, these candidate mutations have to be empirically evaluated. Our initial focus was on Y-F mutations, which were most optimal for AAV2 in a combination of 3 specific capsid residues. In the case of AAV3, a serotype that infects human and primate hepatocytes but is ineffective for mouse, Y-F mutations only have a modest effect, while a combination of S-V and T-V mutations is more optimal [4, 35–37]. Recently, we began to evaluate K-E mutations [19]. Consistent with our published findings using reporter genes, K556E mutation substantially improved FIX expression from an AAV2 vector in hepatic gene transfer. Although not quite as effective as the optimal Y-F triple mutation, it is remarkable that elimination of a single lysine residue can improve efficacy by ~1 log (Figs. 1a, 4a). Unfortunately, it does not appear that this mutation can be combined with others without losing vector yields or infectivity, as changes in capsid structure may impact assembly [19]. More research is required to test whether the K556E mutation indeed reduces AAV2 capsid ubiquitination and to determine the effect on MHC I presentation of capsid antigen.

Previously, we found that the addition of T491V to Y444,500,730F further improved transduction and transgene expression. However, the effect was more modest than the improvement observed for Y444,500,730F compared to single Y-F mutations. Compared to wild-type AAV2, most of the increase in efficacy can be attributed to the Y730F mutation. With each further optimization, improvement becomes more incremental. Although addition of T491V may somewhat further increase translocation of Y444,500,730F vector to the nucleus, this results in no further improvement in systemic FIX expression. The exact mechanism of enhanced gene transfer by specific combination of mutations is unknown. One potential explanation for the improvement in gene transfer with (Y444,500,730F) over the individual Y-F mutations is that the respective localization of these three residues may regulate ubiquitination based on integrative signaling [38].

### Engineering fails to improve the gold standard for mice, AAV8

Next we investigated if engineering could improve murine hepatocyte transduction efficiency of AAV8. Using synonymous Y and T residues in AAV8 we generated two AAV8 variants AAV8-(Y-F)-M3 (275, 447, 733) and AAV8-T494V, which closely mimic previous AAV2 variants. As seen with AAV8K variants [19], both variants failed to improve hFIX expression over wild-type AAV8. AAV8 serotype vectors have exceptional natural tropism for murine hepatocytes in vivo [39]. Unlike AAV2, transduction efficiency is independent of delivery route [40], and upon cellular entry AAV8 viral particles rapidly release their genetic cargo [39]. In contrast, even though AAV2 serotype vectors have been shown to infect nearly all murine hepatocytes, transgene expression is often restricted to a small fraction of hepatocytes [41]. We have shown that this is mediated in part by phosphorylation of the FKBP52 protein and suppression of second-strand synthesis [42, 43]. The kinetics of AAV8 vector uncoating, second strand synthesis, and transgene expression may explain how ssAAV8 vectors bypass this suppression. Our collective data suggest that in mice the ubiquitination-proteasome pathway is not rate limiting for AAV8 gene transfer in mouse liver. This is independently supported by studies that show proteasome inhibitors fail to enhance transgene expression following AAV8 gene transfer [44, 45], although this has been suggested to be dependent on the size of the transgene [45].

### No indication of toxicity from vector infusions in hemophilia B dogs

Of the three dogs injected, only one animal O19 displayed a mild rise in alanine transaminase (ALT) levels, slightly above the upper limit of the reference range provided by the clinical diagnostic laboratory Antech Inc. This elevation was noted prior to vector infusion at baseline and remained in the normal range up to 3 weeks following vector infusion. ALT levels again rose above normal range on day 97 and remained so until the end of the study. Dog O58, which received a threefold higher vector dose compared to O19, maintained normal ALT levels suggesting that this mild elevation in ALT was unrelated to the vector. All animals had a mild reduction in platelet counts during follow-up. Perioperative reductions in platelet counts are probably due to fluid and normal canine plasma administration as well as some consumption at surgical wound sites. Even though some of the values are below the lower end of the reference range, they are not at a level that would be associated with clinical bleeding. Dogs O19 and O58 had mild elevations in hematocrit and hemoglobin levels throughout follow-up and the cause of these mild elevations is not known. Overall the dogs had no clinical concerns related to these small elevations, ate well and maintained body weight throughout the study.

### Limitations of pre-clinical models in general and of the mouse model in particular

**Fig. 5:**
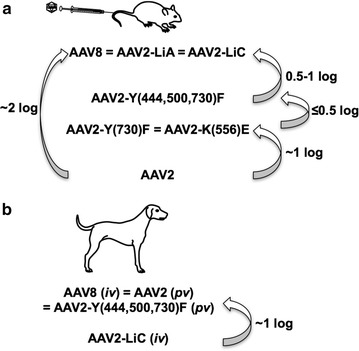
Summary of wild-type and mutant AAV capsids relative liver gene transfer efficiency in **a** mice and **b** dogs. Fold difference is reported on a log scale. 0.5 log is equivalent to ~threefold difference

One of the frustrations with pre-clinical studies on vector development has been that the different animal models may yield substantially differing results on the efficacy of hepatic gene transfer. While AAV2 transduction has been fairly consistent between species, other serotypes vary considerably. For example, AAV8 transduces murine liver 1–2 logs more efficiently than AAV2 (Fig. 5a) [40]. In the canine model, AAV2, 8, and 9 yield similar results, while these serotypes perform quite distinctly in mouse liver [5, 33, 47]. Interestingly, AAV2 and 8 also directed similar FIX levels in humans [11, 12]. It is therefore possible that limitations exist in non-murine species that are not yet understood. In the study presented here, FIX levels achieved in hemophilia B dogs, when adjusted for vector dose, were slightly less for AAV2-(Y-F)-M3 (Fig. 2) compared to historical data on AAV2 (Fig. 5b) [30, 31]. However, when considering that the vector constructs are not entirely identical, were purified with different methods, and were not titered side-by-side, and that the animals are an outbred strain, we conclude that AAV2 and AAV2-(Y-F)-M3 capsids perform similarly in dogs. This contrasts with the 0.5- to 1-log difference in expression seen in mouse models [15, 16].

Both dogs that were treated with AAV2-(Y-F)-M3 were at the approximately the same age and weight at the time of vector injection (Table 2) but received a threefold different vector dose per kg. During the first 2 months after gene transfer, no clear difference in cFIX expression was obvious. However, the animal treated with the lower vector dose (O19), had a hematoma and required a second round of infusion with normal canine plasma at ~2 weeks after gene transfer (it takes 8–17 days for aPTT (activity) and 14–27 days for WBTC to return to baseline following transfusion of cFIX protein [46]). During the third month after gene transfer, WBCT and cFIX activity measurements suggest an ~twofold higher levels of expression in the animal receiving a threefold higher dose (O58). Given biological variability, this outcome is within the expected range, while more animals would be required to determine an exact dose response.

In contrast to the introduction of point mutations to eliminate specific L, Y, T, and S residues, AAV2-LiC capsid had been obtained through a molecular evolution strategy, involving generation of a combinatorial capsid library and repeated in vivo selection in murine liver. Liver-derived FIX expression after peripheral vein injection of AAV2-LiC into different strains of mice is as high as that obtained with AAV8 (Fig. 5a) [20], resulting in induction of robust immune tolerance to FIX in hemophilia B mice (Fig. 3a–c). However, AAV2-LiC performed poorly in the canine model (Fig. 4), with substantially lower efficacy than for example previously reported for AAV8 (Fig. 5b) [33, 48]. We therefore conclude that selection for optimal variants in mouse liver likely results in mouse-specific efficacy that does not translate to other species.

### Implications for future studies and translation

In order to better predict performance of hepatic gene transfer with a particular capsid in human patients, two strategies have been adapted: studies in non-human primates and experiments in immune deficient mice whose livers have been partially reconstituted with human hepatocytes [4, 5, 35, 49, 50]. In the humanized mouse model, we found that different serotypes had highly distinct transduction efficiencies of human hepatocytes, which were often opposite to their relative performance in mouse hepatocytes [5]. An advantage of this model is that human and mouse cell transduction can be directly compared in the same animal. Of the different capsids tested, engineered AAV3 (containing S663V and T492V mutations) outperformed AAV5, 8, and 9 in human hepatocytes [5]. Others who used this model to select for capsids from a “shuffled” library, representing recombined sequences derived from multiple serotypes, also found a capsid primarily consisting of AAV3 sequences to most optimally transduce human hepatocytes [49].

## Conclusions

While only clinical trials can ultimately determine suitability for human gene therapy, it has become clear that results in mouse liver are often not predictive of efficacy in other species or in human liver, so that alternative models need to be used to test rationally designed capsid variants and to select for capsids via directed molecular evolution. New data emerging from ongoing clinical trials evaluating different AAV serotypes will aid in informing which animal model provides the best predictive value for human liver gene transfer efficacy.

## Additional files

**Additional file 1: Table S1.** Clinical chemistry panel for hemophilia B dog O19. Baseline values obtained prior to vector. Pre refers to blood collected on the day of vector administrations and post and onward refer to time points after vector delivery. Low values are colored blue, values within normal ranges are black, and values above normal ranges are red.

**Additional file 2: Table S2.** Clinical chemistry panel for hemophilia B dog O58. Baseline values obtained prior to vector. Day 0 refers to the day of vector administration. Low values are colored blue, values within normal ranges are black, and values above normal ranges are red.

**Additional file 3: Figure S1.** No evidence of bethesda inhibitors in either AAV2-(Y-F)-M3 or AAV2-LiC vector treated hemophilia B dogs. Plasma from selected time points was measured for Bethesda inhibitor titer and are reported as Bethesda units per mL (BU/mL). Data for each animal and the respective vector, delivery route, and dose are represented by a different color (O19-blue, O58-red, Bruce-Green). AAV2-M3 is an abbreviation of AAV2-(Y-F)-M3. *PV* portal vein, *IV* intravenous.

**Additional file 4: Table S3.** Clinical chemistry panel for hemophilia B dog Bruce. Baseline values obtained prior to vector. Day 0 refers to the day of vector administration. Low values are colored blue, values within normal ranges are black, and values above normal ranges are red.

## Abbreviations

hFIX: human factor IX
F9: factor IX gene
cFIX: canine factor IX
FVIII: factor VIII
AAV: adeno-associated virus
ssAAV: single-stranded AAV
MHC I: major histocompatibility complex class I
HB mice: hemophilia B null-mutation mice
BU: Bethesda unit
ELISA: enzyme-linked immunosorbent assay
aPTT: activated partial thromboplastin time
NABs: neutralizing antibodies
WBCT: whole blood clotting time
ALT: alanine transaminase
CBC: complete blood count
