# Supplementary material for: Evaluation of engineered AAV capsids for hepatic factor IX gene transfer in murine and canine models
Source: J Transl Med. 2017 May 1;15:94. doi: 10.1186/s12967-017-1200-1 (PMC5412045; doi:10.1186/s12967-017-1200-1)
Supplement: Supplementary file 1 — Additional file 1: Table S1. Clinical chemistry panel for hemophilia B dog O19. Baseline values obtained prior to vector. Pre refers to blood collected on the day of vector administrations and post and onward refer to time points after vector delivery. Low values are colored blue, values within normal ranges are black, and values above normal ranges are red. [file 12967_2017_1200_MOESM1_ESM.docx]

**Table S1.** Clinical chemistry panel for hemophilia B dog O19. Baseline values obtained prior to vector. Pre refers to blood collected on the day of vector administrations and post and onward refer to time points after vector delivery. Low values are colored blue, values within normal ranges are black, and values above normal ranges are red.

| **Marker (normal range)** | Base | Pre | Post | 1 | 2 | 8 | 15 | 21 | 29 | 43 | 63 | 69 | 77 | 97 | 140 | 175 |
| --- | --- | --- | --- | --- | --- | --- | --- | --- | --- | --- | --- | --- | --- | --- | --- | --- |
| **PLT (200-500 10^3^/mm^3^)** | **166** | **142** | **108** | **132** | **155** | **189** | **166** | 221 | **169** | **174** | **161** | **161** | **155** | **179** | **153** | **178** |
| **WBC (6.0-17.0 10^3^/mm^3^)** | 8.7 | 7.1 | 9.5 | 16.2 | 13.5 | 10.7 | 8.8 | 7.7 | 8 | 8.8 | 8.8 | 8.9 | 8.5 | 8.4 | 7.7 | 9.4 |
| **HCT (37-55%)** | **57.5** | 49.8 | 33.5 | 40.8 | 42.9 | 46.9 | 47 | 44.3 | 52.7 | 48.7 | 53.7 | **55.9** | **56.6** | **57.9** | **59.2** | **58.4** |
| **HGB (12.0-18.0 g/dL)** | **19.5** | **18.5** | 12.5 | 15.2 | 16.1 | 17.3 | 15.6 | 15.4 | **18.3** | **18.2** | **18.9** | **19.4** | **19.6** | 18.6 | 17.8 | 18 |
| **CPK (59-895 U/L)** | 134 | 107 | **956** | 776 | 135 | 181 | 101 | 82 |  |  |  |  |  | 111 | 76 | 111 |
| **ALK PHOS (5-131 U/L)** | 32 | 36 | 30 | 55 | 19 | 38 | 32 | 29 |  |  |  |  |  | 33 | 29 | 37 |
| **ALT (12-118 U/L)** | **128** | 86 | 85 | 89 | 40 | 72 | 92 | 63 |  |  |  |  |  | **157** | **134** | **266** |
| **AST (15-66 U/L)** | 28 | 23 | 50 | 56 | 23 | 32 | 21 | 16 |  |  |  |  |  | 25 | 20 | 39 |
| **Tot Bili (.1-.3 mg/dL)** | 0.1 | 0.1 | 0.1 | 0.1 | 0.1 | 0.1 | 0.1 | 0.2 |  |  |  |  |  | 0.1 | 0.1 | 0.1 |
| **Amyl (290-1125 U/L)** | 652 | 680 | 617 | 478 | **1448** | 742 | 762 | 461 |  |  |  |  |  | 711 | 630 | 664 |
| **Urea N (6.0-25 mg/dL)** | 13 | 15 | 9 | 9 | **38** | 13 | 12 | 10 |  |  |  |  |  | 12 | 12 | 13 |
| **Creat (.5-1.6 mg/dL)** | 0.8 | 0.7 | 0.5 | **0.4** | 1.1 | 0.9 | 0.7 | 0.7 |  |  |  |  |  | 0.7 | 0.7 | 0.7 |
| **BUN/ Creat (4.0-27)** | 16 | 21 | 18 | 23 | **35** | 14 | 17 | 14 |  |  |  |  |  | 17 | 17 | 19 |
| **Total Protein (5.0-7.4 g/L)** | 6.1 | 6.5 | 6.1 | 6.4 | 5.5 | 6.6 | 6.4 | 7.2 |  |  |  |  |  | 6.4 | 6.3 | 6.7 |
| **Alb (2.7-4.4 g/dL)** | 3.6 | 3.5 | 3.5 | 3.6 | **2.6** | 3.8 | 3.6 | 3.8 |  |  |  |  |  | 3.6 | 3.5 | 3.8 |
| **Glu (70-136 mg/dL)** | 108 | 105 | 109 | 108 | 92 | 95 | 94 | 92 |  |  |  |  |  | 84 | 103 | 102 |
| **Chol (92-324 mg/dL)** | 148 | 148 | 150 | 169 | 244 | 147 | 146 | 149 |  |  |  |  |  | 130 | 133 | 134 |
| **Ca+ (8.9-11.4 mg/dL)** | 9.4 | 9.9 | 10 | 9.5 | 10.4 | 10.4 | 10.1 | 10.3 |  |  |  |  |  | 10 | 10 | 10.4 |
| **P (2.5-6.0 mg/dL)** | 3.1 | 3.8 | 4.4 | 3.7 | 4.4 | 4.9 | 3.7 | 5 |  |  |  |  |  | 3.6 | 3.7 | 3.8 |
| **Na+ (139-154 mEq/L)** | 145 | 148 | 148 | 148 | 148 | 147 | 147 | 149 |  |  |  |  |  | 148 | 147 | 148 |
| **K+ (3.6-5.5 mEq/L)** | 4.2 | 4.1 | 3.7 | 4.1 | 5.1 | 5.2 | 4.3 | 4.6 |  |  |  |  |  | 5 | 4.3 | 4.6 |
| **Chl (102-120 mEq/L)** | 111 | 111 | 115 | 114 | 114 | 111 | 111 | 111 |  |  |  |  |  | 111 | 114 | 112 |
| **Alb/ Glob (.8-2.0)** | 1.4 | 1.2 | 1.3 | 1.3 | 0.9 | 1.4 | 1.3 | 1.1 |  |  |  |  |  | 1.3 | 1.3 | 1.3 |
| **Glob (1.6-3.6 g/dL)** | 2.5 | 3 | 2.6 | 2.8 | 2.9 | 2.8 | 2.8 | 3.4 |  |  |  |  |  | 2.8 | 2.8 | 2.9 |
| **Lip (77-695 U/L)** | 229 | 247 | 284 | 172 | 306 | 93 | 175 | 127 |  |  |  |  |  | 240 | 174 | 230 |
| **Triglyc (29-291 mg/dL)** | 52 | 41 | **27** | 36 | 83 | 35 | 41 | 33 |  |  |  |  |  | 37 | 37 | 36 |
| **Mg+ (1.5-2.5 mEq/L)** | 1.5 | 1.6 | 1.5 | 1.7 | 1.6 | 1.9 | 1.6 | 1.9 |  |  |  |  |  | 1.7 | 1.7 | 1.8 |
