# Supplementary material for: Evaluation of engineered AAV capsids for hepatic factor IX gene transfer in murine and canine models
Source: J Transl Med. 2017 May 1;15:94. doi: 10.1186/s12967-017-1200-1 (PMC5412045; doi:10.1186/s12967-017-1200-1)
Supplement: Supplementary file 2 — Additional file 2: Table S2. Clinical chemistry panel for hemophilia B dog O58. Baseline values obtained prior to vector. Day 0 refers to the day of vector administration. Low values are colored blue, values within normal ranges are black, and values above normal ranges are red. [file 12967_2017_1200_MOESM2_ESM.docx]

**Table S2.** Clinical chemistry panel for hemophilia B dog O58. Baseline values obtained prior to vector. Day 0 refers to the day of vector administration. Low values are colored blue, values within normal ranges are black, and values above normal ranges are red.

| **Marker (normal range)** | Base | 0 | 1 | 2 | 7 | 14 | 22 | 27 | 41 | 50 | 65 | 71 | 78 | 85 | 92 | 99 |
| --- | --- | --- | --- | --- | --- | --- | --- | --- | --- | --- | --- | --- | --- | --- | --- | --- |
| **PLT (200-500 10^3^/mm^3^)** | **186** | **157** | **135** | **127** | **179** | 212 | **196** | 216 | 229 | **170** | **197** | **194** | **195** | **190** | 213 | 221 |
| **WBC (6.0-17.0 10^3^/mm^3^)** | 6.4 | 12.5 | 14.8 | 12.3 | 11.6 | 10.9 | 8 | 8.4 | 8.8 | 7.6 | 9.2 | 11.2 | 8.3 | 9.9 | 10.9 | 8.9 |
| **HCT (37-55%)** | **57.9** | 49.5 | 43.8 | 41.4 | 46.5 | **55.2** | **61.3** | **61.1** | **63.5** | **56.5** | **57.7** | **58.8** | 52.5 | **56.3** | **59.5** | **59** |
| **HGB (12.0-18.0 g/dL)** | **18.4** | 16.1 | 14.3 | 13.8 | 14.6 | 18 | **19.6** | **18.8** | **19.3** | **18.3** | **18.6** | **18.3** | 17.1 | **18.1** | **18.5** | 18 |
| **CPK (59-895 U/L)** |  | 165 | 124 |  | 203 | 127 |  |  |  |  |  |  |  |  |  | 176 |
| **ALK PHOS (5-131 U/L)** |  | 19 | 33 |  | 26 | 22 |  |  |  |  |  |  |  |  |  | 14 |
| **ALT (12-118 U/L)** |  | 70 | 92 |  | 62 | 63 |  |  |  |  |  |  |  |  |  | 57 |
| **AST (15-66 U/L)** |  | 39 | 27 |  | 31 | 20 |  |  |  |  |  |  |  |  |  | 30 |
| **Tot Bili (.1-.3 mg/dL)** |  | 0.2 | 0.1 |  | 0.1 | 0.1 |  |  |  |  |  |  |  |  |  | 0.1 |
| **Amyl (290-1125 U/L)** |  | 806 | 616 |  | 438 | 628 |  |  |  |  |  |  |  |  |  | 458 |
| **Urea N (6.0-25 mg/dL)** |  | 9 | 8 |  | 8 | 10 |  |  |  |  |  |  |  |  |  | 10 |
| **Creat (.5-1.6 mg/dL)** |  | 0.5 | 0.5 |  | 0.5 | 0.6 |  |  |  |  |  |  |  |  |  | 0.5 |
| **BUN/ Creat (4.0-27)** |  | 18 | 16 |  | 16 | 17 |  |  |  |  |  |  |  |  |  | 20 |
| **Total Protein (5.0-7.4 g/L)** |  | 6.3 | 6.7 |  | 6.9 | 6.8 |  |  |  |  |  |  |  |  |  | 5.7 |
| **Alb (2.7-4.4 g/dL)** |  | 3.7 | 3.8 |  | 3.9 | 4 |  |  |  |  |  |  |  |  |  | 3.5 |
| **Glu (70-136 mg/dL)** |  | 95 | 97 |  | 100 | 116 |  |  |  |  |  |  |  |  |  | 98 |
| **Chol (92-324 mg/dL)** |  | 161 | 205 |  | 223 | 139 |  |  |  |  |  |  |  |  |  | 141 |
| **Ca+ (8.9-11.4 mg/dL)** |  | 10.2 | 9.6 |  | 10.4 | 10.5 |  |  |  |  |  |  |  |  |  | 9.2 |
| **P (2.5-6.0 mg/dL)** |  | 5.7 | 4.9 |  | 5.4 | 4.6 |  |  |  |  |  |  |  |  |  | 3.3 |
| **Na+ (139-154 mEq/L)** |  | 150 | 148 |  | 149 | 145 |  |  |  |  |  |  |  |  |  | 147 |
| **K+ (3.6-5.5 mEq/L)** |  | 3.9 | 3.7 |  | 4.4 | 4.2 |  |  |  |  |  |  |  |  |  | 4.2 |
| **Chl (102-120 mEq/L)** |  | 114 | 112 |  | 108 | 110 |  |  |  |  |  |  |  |  |  | 113 |
| **Alb/ Glob (.8-2.0)** |  | 1.4 | 1.3 |  | 1.3 | 1.4 |  |  |  |  |  |  |  |  |  | 1.6 |
| **Glob (1.6-3.6 g/dL)** |  | 2.6 | 2.9 |  | 3 | 2.8 |  |  |  |  |  |  |  |  |  | 2.2 |
| **Lip (77-695 U/L)** |  | 427 | 211 |  | 78 | 218 |  |  |  |  |  |  |  |  |  | 148 |
| **Triglyc (29-291 mg/dL)** |  | **24** | 33 |  | 46 | 40 |  |  |  |  |  |  |  |  |  | 31 |
| **Mg+ (1.5-2.5 mEq/L)** |  | **1.3** | **1.4** |  | 1.5 | 1.6 |  |  |  |  |  |  |  |  |  | **1.3** |
