# Supplementary figures and images for: Evaluation of engineered AAV capsids for hepatic factor IX gene transfer in murine and canine models
Source: J Transl Med. 2017 May 1;15:94. doi: 10.1186/s12967-017-1200-1 (PMC5412045; doi:10.1186/s12967-017-1200-1)

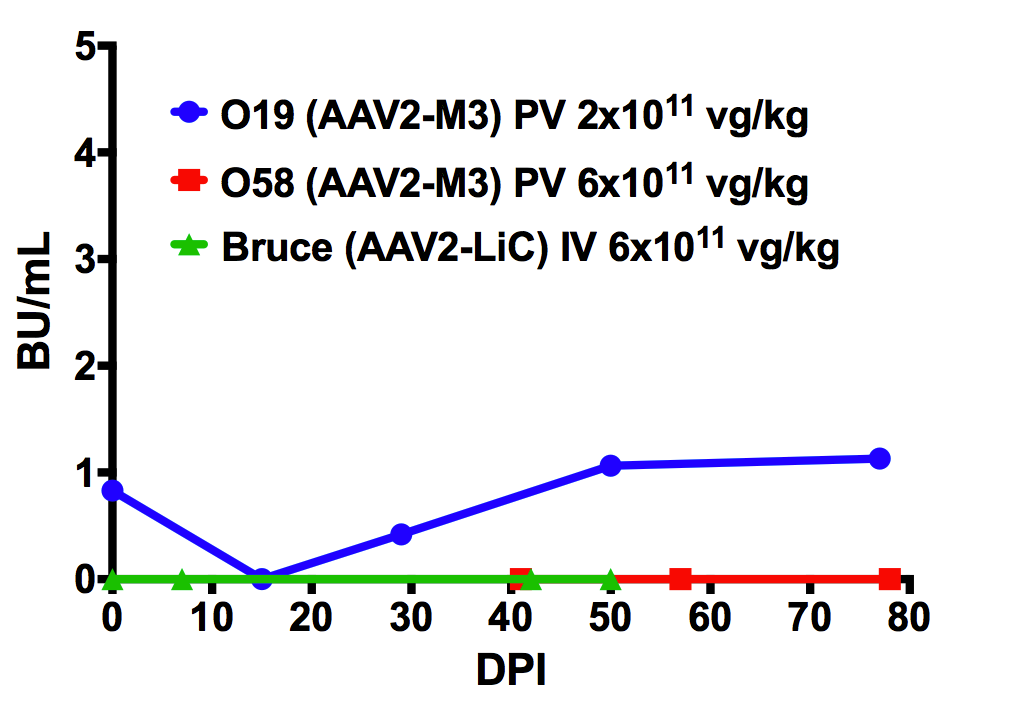

Supplement: Supplementary file 3 — Additional file 3: Figure S1. No evidence of bethesda inhibitors in either AAV2-(Y-F)-M3 or AAV2-LiC vector treated hemophilia B dogs. Plasma from selected time points was measured for Bethesda inhibitor titer and are reported as Bethesda units per mL (BU/mL). Data for each animal and the respective vector, delivery route, and dose are represented by a different color (O19-blue, O58-red, Bruce-Green). AAV2-M3 is an abbreviation of AAV2-(Y-F)-M3. PV portal vein, IV intravenous. [file 12967_2017_1200_MOESM3_ESM.tiff]
