# Supplementary material for: Evaluation of engineered AAV capsids for hepatic factor IX gene transfer in murine and canine models
Source: J Transl Med. 2017 May 1;15:94. doi: 10.1186/s12967-017-1200-1 (PMC5412045; doi:10.1186/s12967-017-1200-1)
Supplement: Supplementary file 4 — Additional file 4: Table S3. Clinical chemistry panel for hemophilia B dog Bruce. Baseline values obtained prior to vector. Day 0 refers to the day of vector administration. Low values are colored blue, values within normal ranges are black, and values above normal ranges are red. [file 12967_2017_1200_MOESM4_ESM.docx]

**Table S3.** Clinical chemistry panel for hemophilia B dog Bruce. Baseline values obtained prior to vector. Day 0 refers to the day of vector administration. Low values are colored blue, values within normal ranges are black, and values above normal ranges are red.

| **Marker (normal range)** | Pre | 0 | 1 | 2 | 3 | 7 | 15 | 21 | 35 | 42 | 56 | 63 | 70 | 78 | 86 | 92 | 99 |
| --- | --- | --- | --- | --- | --- | --- | --- | --- | --- | --- | --- | --- | --- | --- | --- | --- | --- |
| **PLT (200-500 10^3^/mm^3^)** | 206 | **180** | 209 | **190** | **198** | 196 | **179** | 213 | 214 | 200 | 213 | **176** | **198** | 203 | **199** | **190** | **194** |
| **WBC (6.0-17.0 10^3^/mm^3^)** | 10.9 | 10 | 8.8 | 10.8 | 11.1 | 9.3 | 8.6 | 10 | 8 | 8.7 | 12.2 | 9 | 11.1 | 11.5 | 10.4 | 7.7 | 13.6 |
| **HCT (37-55%)** | **57.1** | 49.2 | 52.7 | 50.4 | 48.5 | 52.9 | 50.9 | 55.7 | 52.1 | 49.9 | 49.1 | 52.2 | 54.1 | 53.4 | 54 | 55 | 54.9 |
| **HGB (12.0-18.0 g/dL)** | 18 | 15.5 | 16.4 | 15.4 | 15.1 | 16.5 | 16.1 | 17.4 | 16.2 | 15.6 | 15.1 | 16.3 | 17.1 | 17 | 17.2 | 17 | 17.8 |
| **CPK (59-895 U/L)** | 111 | 87 | 62 |  |  | 104 |  |  |  |  |  |  |  |  |  |  | 113 |
| **ALK PHOS (5-131 U/L)** | 59 | 49 | 48 |  |  | 45 |  |  |  |  |  |  |  |  |  |  | 46 |
| **ALT (12-118 U/L)** | 44 | 48 | 50 |  |  | 48 |  |  |  |  |  |  |  |  |  |  | 40 |
| **AST (15-66 U/L)** | 20 | 20 | 18 |  |  | 23 |  |  |  |  |  |  |  |  |  |  | 21 |
| **Tot Bili (.1-.3 mg/dL)** | 0.1 | 0.1 | 0.1 |  |  | 0.1 |  |  |  |  |  |  |  |  |  |  | 0.1 |
| **Amyl (290-1125 U/L)** | 523 | 483 | 409 |  |  | 519 |  |  |  |  |  |  |  |  |  |  | 575 |
| **Urea N (6.0-25 mg/dL)** | 15 | 13 | 17 |  |  | 13 |  |  |  |  |  |  |  |  |  |  | 15 |
| **Creat (.5-1.6 mg/dL)** | 0.7 | 0.5 | 0.6 |  |  | 0.6 |  |  |  |  |  |  |  |  |  |  | 0.6 |
| **BUN/ Creat (4.0-27)** | 21 | 26 | **28** |  |  | 22 |  |  |  |  |  |  |  |  |  |  | 25 |
| **Total Protein (5.0-7.4 g/L)** | 6.6 | 5.9 | 5.9 |  |  | 5.6 |  |  |  |  |  |  |  |  |  |  | 6 |
| **Alb (2.7-4.4 g/dL)** | 3.4 | 3.4 | 3.3 |  |  | 3.4 |  |  |  |  |  |  |  |  |  |  | 3.2 |
| **Glu (70-136 mg/dL)** | 89 | 103 | 96 |  |  | 105 |  |  |  |  |  |  |  |  |  |  | 94 |
| **Chol (92-324 mg/dL)** | 160 | 139 | 150 |  |  | 138 |  |  |  |  |  |  |  |  |  |  | 138 |
| **Ca+ (8.9-11.4 mg/dL)** | 9.9 | 9.7 | 10 |  |  | 9.8 |  |  |  |  |  |  |  |  |  |  | 9.5 |
| **P (2.5-6.0 mg/dL)** | 3.6 | 4.7 | 4.6 |  |  | 4.3 |  |  |  |  |  |  |  |  |  |  | 4.2 |
| **Na+ (139-154 mEq/L)** | 149 | 150 | 150 |  |  | 147 |  |  |  |  |  |  |  |  |  |  | 147 |
| **K+ (3.6-5.5 mEq/L)** | 4.1 | 4.2 | 4.2 |  |  | 4.3 |  |  |  |  |  |  |  |  |  |  | 4.3 |
| **Chl (102-120 mEq/L)** | 113 | 115 | 116 |  |  | 113 |  |  |  |  |  |  |  |  |  |  | 112 |
| **Alb/ Glob (.8-2.0)** | 1.1 | 1.4 | 1.3 |  |  | 1.5 |  |  |  |  |  |  |  |  |  |  | 1.1 |
| **Glob (1.6-3.6 g/dL)** | 3.2 | 2.5 | 2.6 |  |  | 2.2 |  |  |  |  |  |  |  |  |  |  | 2.8 |
| **Lip (77-695 U/L)** | 151 | 170 | 178 |  |  | 139 |  |  |  |  |  |  |  |  |  |  | 128 |
| **Triglyc (29-291 mg/dL)** | 41 | 31 | 50 |  |  | 39 |  |  |  |  |  |  |  |  |  |  | 45 |
| **Mg+ (1.5-2.5 mEq/L)** | **1.4** | **1.3** | 1.5 |  |  | **1.4** |  |  |  |  |  |  |  |  |  |  | **1.4** |
